# Osteochondritis Dissecans of the Knee Associated With Mechanical Overload

**DOI:** 10.1177/03635465231211497

**Published:** 2024-01-02

**Authors:** Goran S. van der Weiden, Stijn van Cruchten, Nienke van Egmond, Simon C. Mastbergen, Martin Husen, Daniël B.F. Saris, Roel J.H. Custers

**Affiliations:** * Rheumatology & Clinical Immunology, University Medical Center Utrecht, Utrecht University, Utrecht, the Netherlands; † Department of Orthopedic Surgery, University Medical Center Utrecht, Utrecht University, Utrecht, the Netherlands; ‡ Department of Orthopedic Surgery, Mayo Clinic, Rochester, Minnesota, USA; § Department of Orthopedic Surgery, University Hospital Heidelberg, Heidelberg, Germany; Investigation performed at University Medical Center Utrecht, Utrecht University, Utrecht, the Netherlands

**Keywords:** osteochondritis dissecans, OCD, etiology, demographics, mechanical overload, knee joint

## Abstract

**Background:**

Osteochondritis dissecans (OCD) of the knee is a rare but potentially incapacitating disorder in which subchondral bone detaches, leading to an osteochondral fragment that can become unstable and progress into a loose body. The exact cause is unknown, although several biological and mechanical factors have been described.

**Purpose:**

To provide insight into epidemiological data of a large cohort of patients affected by OCD of the knee and to identify potential factors contributing to the cause of this disorder.

**Study Design:**

Cross-sectional study; Level of evidence, 3.

**Methods:**

A total of 236 patients (259 knees) affected by OCD were included in our Knee Registry (2005-2022) and retrospectively analyzed. Patient characteristics were extracted from the medical records. Location and International Cartilage Regeneration & Joint Preservation Society grade (1-4) of OCD were assessed using magnetic resonance imaging. If available, a full-leg standing radiograph was used to assess alignment. Additionally, a statistical scoring system for instability risk was created.

**Results:**

A total of 263 OCD lesions were identified in 259 knees, 66.2% on the medial femoral condyle (MFC), 26.6% on the lateral femoral condyle (LFC), 3.8% on the trochlea, 2.7% on the patella, and 0.8% on the lateral tibia plateau. Male patients made up 57.6% of the sample, which had a mean age of 21.8 years. A very high percentage of patients (77.1%; n = 182) practiced sports, of whom 67.6% (n = 123) were engaged in high-impact sports. The location of the OCD lesions and the leg alignment (n = 110) were significantly correlated: MFC lesions were associated with more varus than valgus alignment (47.5% vs 11.3%) and patients with LFC lesions had more valgus than varus alignment (46.7% vs 20.0%; *P* = .002). Based on age, smoking, sports activity, and preceding trauma, a multivariable scoring system (0-11 points) was created. An increased risk of lesion instability was associated with an increased score: 29.0% at 0 points and 97.0% at 11 points.

**Conclusion:**

This study provides detailed epidemiological data for 236 patients affected by OCD of the knee. Older age, smoking, inactivity, and preceding trauma were predictive for instability of OCD lesions. There was an association between OCD of the MFC and varus malalignment and between OCD of the LFC and valgus malalignment. This finding, in combination with the high percentage of patients practicing high-impact sports, suggests an important role for mechanical overload in the pathogenesis of OCD.

Osteochondritis dissecans (OCD) is a rare joint disorder leading to the partial separation of a subchondral bone fragment from its bony bed. This can, along with the overlying cartilage, become unstable and even progress into a loose body in the joint space.^16^ Although OCD affects most larger joints, such as the hip, ankle, and elbow, the knee is the most frequently affected joint in the pediatric population.^18^ The most commonly affected location within the knee joint is the medial femoral condyle (MFC), followed by the lateral femoral condyle (LFC) and the patella.^3,39^ OCD of the knee joint occurs mostly in young, active people^12^; the highest incidence is found in patients 12 to 19 years old.^8^ Reported OCD incidence has increased substantially in the last century^26^ to 6 to 11 per 100,000 persons.^28,35^

König^27^ first named the disorder in 1888, originally hypothesizing an inflammatory origin. Hence arose the terms *osteochondritis*, to refer to inflammation of the osteochondral surface, and *dissecans*, derived from the Latin word for separation: *dissec*. Although subsequent histologic studies have shown focal osseous necrosis, with no evidence supporting an inflammatory cause, the name has persisted.^5,21,42^ Several more likely potential etiological factors have been described since then, including repetitive microtrauma,^10,29^ vascular insufficiency,^37^ obesity,^25^ and hereditary factors^20,38^ in pediatric patients and associated mechanical axis deviations in both pediatric and adult patients.^7,19,23^ The exact cause remains unknown,^16^ due to high variability and limited cohort sizes of studies, and none of the proposed etiological factors are indisputable.

OCD lesions present in a highly variable manner. Although stable OCD lesions are often asymptomatic, they can also lead to vague complaints and poorly localized knee pain that is exacerbated by exercise. This can lead to considerable delay of diagnosis.^9^ Unstable lesions and loose fragments may cause stiffness, joint effusion, instability, and mechanical symptoms such as locking sensations and may require surgical treatment.^1,13,26^ Skeletal maturity is associated with a significantly decreased success rate for nonoperative treatment when compared with skeletal immaturity.^9,18,44^ The arthroscopic International Cartilage Regeneration & Joint Preservation Society (ICRS) grading is historically often used to assess stability of the OCD, although noninvasive imaging such as radiography and magnetic resonance imaging (MRI) are usually used initially.^4^

The department of orthopaedic surgery of our institution is an International Expertise Center for OCD (European Reference Network) of the knee, providing the opportunity to investigate one of the largest cohort of patients to date. The purpose of this study was to provide epidemiological data of patients affected by OCD of the knee and to identify possible risk factors contributing to the cause of the disorder. Additionally, we aimed to develop a model to predict the stability of OCD lesions in the prehospital setting.

## Methods

We performed a retrospective cohort study using data from the Knee Registry, a database containing patients with knee disorders who visited the Orthopedics Department of the UMC Utrecht. All patients gave written informed consent before inclusion in this study (IRB No. 17-005). The institutional medical records database was searched to identify patients diagnosed with OCD of the knee joint between January 2005 and June 2022. All patients with confirmed OCD were included, independent of the received treatment. Exclusion criteria consisted of osteochondral defects not as a consequence of osteochondritis dissecans, insufficient documentation or absent medical imaging to verify diagnosis, and absence of written consent to participate in the Knee Registry.

Patient data collected from medical charts included age, sex, side affected, age at diagnosis, age at start of symptoms, body mass index (BMI), laterality, preceding trauma, frequency and impact level of practiced sports, smoking habits, and relevant medical history. Past medical history was deemed relevant if known to be a risk factor for osteonecrosis, OCD, or congenital musculoskeletal disorders. Sport impact level was categorized as low impact (swimming, cycling, low-impact combat sports, low-impact dancing) and high impact (running, ball sports, high-impact combat sports, high-impact dancing).

**Figure 1.**
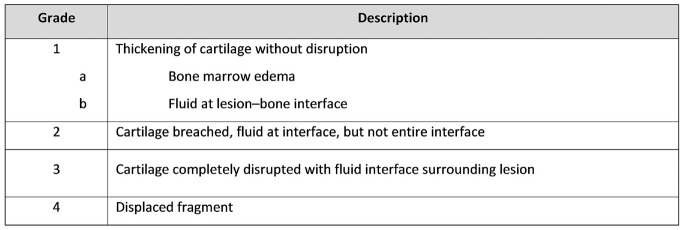
International Cartilage Regeneration & Joint Preservation Society Magnetic Resonance Imaging grading system for osteochondritis dissecans based on the grading system as described by Dipaola et al.^14^

Skeletal maturity was assessed on standard knee radiographs and MRI scans at the time of OCD diagnosis: patients with open or closing physes were considered skeletally immature, and patients with closed growth plates were considered skeletally mature. Visibility of the OCD on the radiograph was assessed when available. OCD lesions were graded on MRI scans using the ICRS MRI grading system^17^ based on the grading system described by Dipaola et al^14^ (Figure 1). Lesions grade 1 or 2 are considered stable, whereas grade 3 or 4 lesions are unstable. The maximum width, maximum length, and maximum thickness were measured on MRI scans. The volume of the OCD lesions was calculated assuming an ellipsoid shape, previously described for other medical applications^24,30^:

$$\mathrm{Volume}\;\mathrm{in}\;{\mathrm{cm}}^{3}=\frac{4}{3}*\pi *\frac{a}{2}*\frac{b}{2}*\frac{c}{2},$$

where *a* is maximum width, *b* is maximum length, and *c* is maximum thickness.

Assessment was performed by 2 observers (G.S.v.W. and S.v.C.) who were blinded to the source of the imaging. A consensus-based scoring system was used, with potential discrepancies to be decided by a third observer (R.J.H.C.), although this was not necessary at any moment.

**Figure 2.**
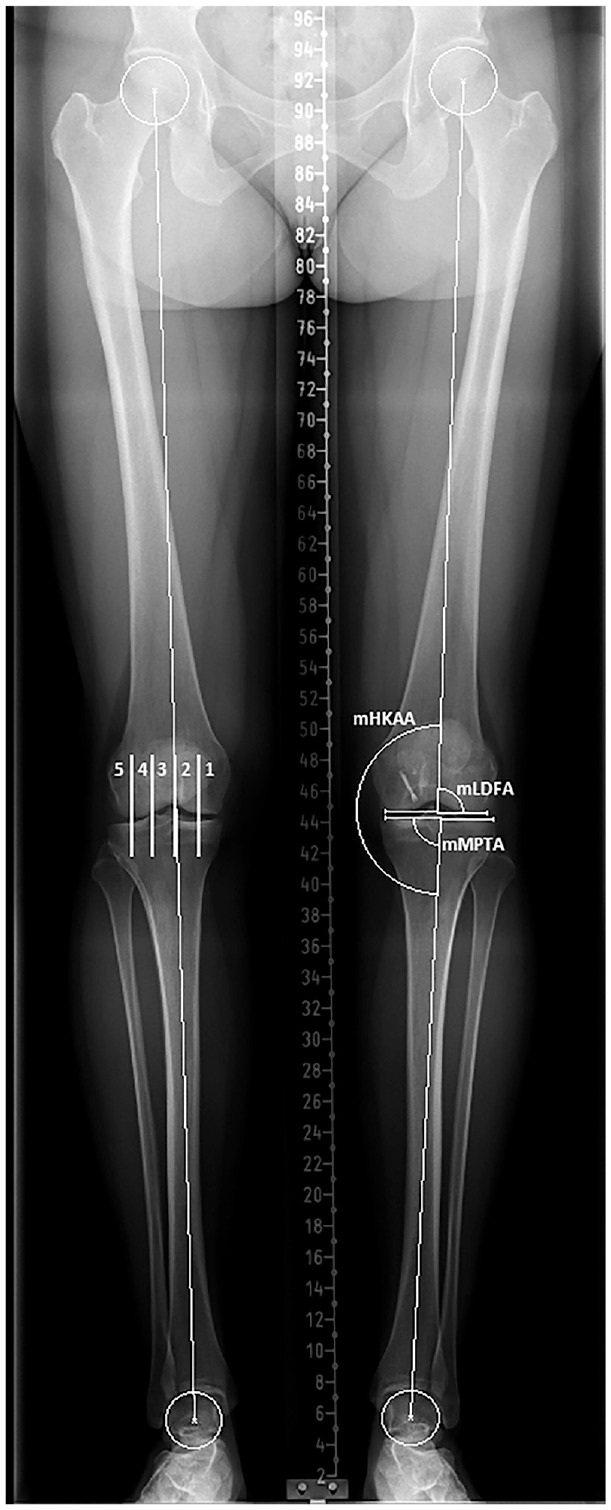
The mechanical axis for each patient was categorized as varus (1 or 2), neutral (3), or valgus (4 or 5) alignment based on the area of the knee that the mechanical axis line intersected, as described by Cahill and Berg.^10^ mHKAA, mechanical hip-knee-ankle angle; mLDFA, mechanical lateral distal femoral angle; mMPTA, mechanical medial proximal tibial angle.

When available, full-length standing anteroposterior radiographs of the lower extremities were assessed. The mechanical axis of the lower extremity, hip-knee-ankle angle (HKAA), mechanical lateral distal femoral angle, mechanical medial proximal tibial angle, joint line convergence angle, and Mikulicz lines were measured using a digital picture archiving and communication system.^11,34,41^ The mechanical axis for each patient was categorized as varus, neutral, or valgus alignment based on the area of the knee described by Cahill and Berg^10^ that the mechanical axis line intersected^19^ (Figure 2).

### Statistical Analysis

Descriptive statistics were presented as number of cases and percentages for dichotomous variables and as mean with standard deviation for continuous variables. Independent *t* test was used for continuous variables; chi-square test and Fisher exact test were used for categorical variables. Correlation between lesion volume, ICRS grade, and degree of mechanical axis deviation was assessed using the Pearson correlation coefficient. All statistical tests were 2-sided, and *P* values <.05 were considered statistically significant.

A statistical scoring system to predict stability of the OCD lesions on MRI scans was created using the methods previously described by Sullivan et al.^43^ Lesions were randomly divided into two-thirds and one-thirds groups using a random number generator. The 2/3 group was used to develop the model, and the 1/3 group was used to validate the model. Readily available variables in a prehospital setting were used, including age, sex, BMI, relevant medical history, smoking status, sport impact, and preceding trauma. All relevant variables were entered into the regression model, with a maximum of variables equal to ≥10 events per variable criterion.^31,36^ Variables were removed from the model if *P*≥ .2. Area under the receiver operating characteristic curve (AUC) was used to assess diagnostic accuracy of the model. For all statistical tests, IBM SPSS Statistics version 29.0.0 was used.

## Results

Of the 344 identified patients, 236 gave written informed consent to be included in the study cohort. These patients had a total of 259 affected knees, of which 91 were skeletally immature and 168 were skeletally mature. Patient characteristics are presented in Table 1. Skeletally immature patients were significantly younger, as expected, and had a lower BMI, higher percentage of sport participation, and higher percentage of high-impact sports participation. Skeletally mature patients had significantly higher ICRS grade OCD lesions on MRI scans. We found no significant differences in sex, side, location of OCD, preceding trauma, relevant medical history, mechanical alignment, and volume of OCD lesion between skeletally immature and mature patients.

**Table 1:** Patient Characteristics^*a*^

Characteristics	Skeletally Immature	Skeletally Mature	*P*	Total
Patients, n	79	157		236
Knees, n	91	168		259
Male	45 (57.0)	91 (58.0)	.890	136 (57.6)
Age at presentation, y	15.7 ± 4.0	24.9 ± 7.8	**<.001**	21.8 ± 8.0
Time to presentation, y	2.7 ± 2.5	3.6 ± 3.9	.057	3.3 ± 3.5
Body mass index	21.8 ± 3.8	24.1 ± 3.8	**<.001**	23.4 ± 3.95
Side			.096	
Left	33 (41.8)	81 (51.6)		114 (48.3)
Right	34 (43.0)	65 (41.4)		99 (41.9)
Bilateral	12 (15.2)	11 (7.0)		23 (9.8)
Location, n (%) of knees			.264	
MFC	58 (63.7)	114 (67.9)		172 (66.4)
LFC	22 (24.2)	44 (26.2)		66 (25.5)
Trochlea	5 (5.5)	4 (2.4)		9 (3.5)
Patella	2 (2.2)	5 (3.0)		7 (2.7)
LTP	1 (1.1)	0		1 (0.4)
Multiple	3 (3.3)	1 (0.6)		4 (1.5)
MFC and LFC	2 (66.7)			
LFC and trochlea	1 (33.3)			
LFC and LTP		1 (100)		
Previous trauma, n (%) of knees	21 (23.1)	34 (20.2)	.634	55 (21.2)
Smoking	2 (2.5)	18 (11.5)	**.023**	20 (8.5)
Practicing sports weekly	68 (86.1)	114 (72.6)	**.022**	182 (77.1)
Practicing high-impact sports	57 (83.8)	66 (57.9)	**<.001**	123 (67.6)
Relevant medical history			.347	
Methotrexate/corticosteroid	2 (2.5)	1 (0.6)		3 (1.3)
Sepsis	0	1 (0.6)		1 (0.4)
Congenital musculoskeletal disorder	1 (1.3)	4 (2.5)		5 (2.1)
Operation index knee	0	1 (0.6)		1 (0.4)
Perthes	0	1 (0.6)		1 (0.4)
Acute lymphoblastic leukemia	0	1 (0.6)		1 (0.4)
Juvenile idiopathic arthritis	1 (1.3)	0		1 (0.4)
Mechanical alignment	(n = 28)	(n = 85)	.215	(n = 113)
Neutral	14 (50.0)	31 (36.5)		45 (39.8)
Varus	7 (25.0)	37 (43.5)		44 (39.0)
Valgus	7 (25.0)	17 (20.0)		24 (21.2)
Associated location—mechanical alignment	9 (36.0) (n = 25)	43 (50.6) (n = 85)	.256	52 (47.3) (n = 110)
MFC varus	6 (31.6) (n = 19)	32 (52.5) (n = 61)		38 (47.5) (n = 80)
LFC valgus	3 (50.0) (n = 6)	11 (45.8) (n = 24)		14 (46.7) (n = 30)
Visibility on first radiograph	(n = 88)	(n = 154)	.411	(n = 242)
Visible	80 (90.9)	133 (86.4)		213 (88.0)
Not visible	8 (9.1)	21 (13.6)		29 (12.0)
ICRS grade	(n = 78)	(n = 132)	**<.001**	(n = 210)
1	27 (34.6)	9 (6.8)		36 (17.1)
2	26 (33.3)	39 (29.5)		65 (31.0)
3	15 (19.3)	45 (34.1)		60 (28.6)
4	10 (12.8)	39 (29.5)		49 (23.3)
Size, cm^3^	1.12 ± 1.26	1.22 ± 0.78	.549	1.18 ± 0.98

a Values are expressed as n (%) or mean ± SD unless otherwise noted; n (%) refers to number of affected knees unless otherwise noted. Boldface indicates statistical significance. ICRS, International Cartilage Regeneration & Joint Preservation Society; LFC, lateral femoral condyle; LTP, lateral tibia plateau; MFC, medial femoral condyle.

In total, 101 patients (110 affected knees) with OCD lesions on the MFC (80 affected knees) or LFC (30 affected knees) had available full-length standing anteroposterior radiographs. In this subpopulation, patient characteristics did not significantly differ compared with the total population. Evaluations of the mechanical axis between OCD lesions of the MFC and LFC are presented in Table 2. No significant differences were seen in ICRS grade, lesion volume, mechanical medial proximal tibial angle, or joint line convergence angle. Patients with OCD lesions on the MFC were significantly more likely to have varus alignment, and patients with OCD lesions on the LFC were significantly more likely to have valgus alignment (*P* = .002). Similarly, mean mHKAA for OCD lesions on the MFC was 1.79°± 3.20° varus compared with 1.43°± 3.52° valgus in OCD lesions on the LFC (*P* < .001) (Table 2). Patients with OCD lesions on the MFC had a statistically significantly higher mechanical lateral distal femoral angle (88.5°± 2.7°) compared with patients with OCD lesions on the LFC (86.1°± 2.7°) (*P* < .001). Patients with OCD lesions on the MFC had a significantly lower Mikulicz angle (42.3°± 16.3°) compared with patients with OCD lesions on the LFC (53.7°± 16.7°) (*P* = .002).

**Table 2:** Evaluations of Mechanical Axis (Patients With Available Leg Axis)^*a*^

Characteristic	Medial Femoral Condyle	Lateral Femoral Condyle	*P*
Patients, n	72	29	
Knees, n	80	30	
Mechanical alignment			**.002**
Neutral	33 (41.3)	10 (33.3)	
Varus	38 (47.5)	6 (20.0)	
Valgus	9 (11.3)	14 (46.7)	
mHKAA, deg	1.79 ± 3.20 varus	1.43 ± 3.52 valgus	**<.001**
mFTA, deg	1.73 ± 3.08 varus	1.10 ± 3.50 valgus	**<.001**
mLDFA, deg	88.5 ± 2.7	86.1 ± 2.7	**<.001**
mMPTA, deg	88.1 ± 2.5	87.4 ± 2.9	.206
Mikulicz angle, deg	42.3 ± 16.3	53.7 ± 16.7	**.002**
JLCA, deg	1.6 ± 1.2	1.5 ± 1.5	.953
Skeletally mature	61 (76.3)	24 (80)	**.023**
ICRS grade	(n = 65)	(n = 24)	.066
1	8 (12.3)	3 (10.0)	
2	18 (27.7)	9 (30.0)	
3	24 (36.9)	6 (20.0)	
4	15 (23.1)	6 (20.0)	
Size, cm^3^	1.41 ± 1.29 (n = 67)	1.18 ± 0.79 (n = 26)	.407

a Values are expressed as n (%) or mean ± SD unless otherwise indicated; n (%) refers to number of affected kneespatients unless otherwise noted. Boldface indicates statistical significance. ICRS, International Cartilage Regeneration & Joint Preservation Society; JLCA, joint line convergence angle; mFTA, mechanical femorotibial angle; mHKAA, mechanical hip-knee-ankle angle; mLDFA, mechanical lateral distal femoral angle; mMPTA, mechanical medial proximal tibial angle.

Patients were stratified to in situ lesions (ICRS grade 1-3) and displaced lesions (ICRS grade 4), as presented in Table 3. In patients with OCD lesions on the MFC, in situ and displaced lesions showed similar rates of varus alignment, 44.0% versus 53.3%, respectively (*P* = .754). Mean mHKAA for in situ lesions was 1.42°± 2.52° varus compared with 3.08°± 4.78° varus in displaced OCD lesions (*P* = .214). In patients with OCD lesions on the LFC, in situ lesions were more likely to have valgus alignment compared with displaced lesions, 61.1% versus 33.3%, respectively (*P* = .364). Mean mHKAA for in situ lesions was 2.14°± 4.24° valgus compared with 0.45°± 1.58° valgus in displaced OCD lesions (*P* = .356).

**Table 3:** Evaluations of Mechanical Axis (Stratified for In Situ or Displaced Fragments)^*a*^

Characteristic	Medial Femoral Condyle			Lateral Femoral Condyle		
	In Situ	Displaced	*P*	In Situ	Displaced	*P*
Patients, n	47	14		18	5	
Knees, n	50	15		18	6	
Mechanical alignment			.754			.364
Neutral	22 (44.0)	6 (40.0)		5 (27.8)	2 (33.3)	
Varus	22 (44.0)	8 (53.3)		2 (11.1)	2 (33.3)	
Valgus	6 (12.0)	1 (6.7)		11 (61.1)	2 (33.3)	
mHKAA, deg	1.42 ± 2.52 varus	3.08 ± 4.78 varus	.214	2.14 ± 4.24 valgus	0.45 ± 1.58 valgus	.356
mFTA, deg	1.26 ± 2.52 varus	3.60 ± 4.09 varus	**.050**	1.89 ± 4.19 valgus	0.00 ± 1.55 neutral	.297
mLDFA, deg	88.2 ± 2.3	88.3 ± 3.4	.931	85.8 ± 2.5	87.2 ± 3.0	.271
mMPTA, deg	88.5 ± 2.5	86.7 ± 2.5	**.017**	87.4 ± 3.2	88.0 ± 3.1	.712
Mikulicz angle, deg	44.5 ± 13.6	36.3 ± 23.4	.210	57.4 ± 19.7	50.0 ± 7.5	.382
JLCA, deg	1.8 ± 1.3	1.4 ± 1.1	.337	1.4 ± 1.9	1.7 ± 0.8	.784
Skeletally mature	35 (70.0)	13 (87)	.317	13 (72)	6 (100)	.280
Size, cm^3^	1.43 ± 1.44	1.21 ± 0.64	.562	1.20 ± 0.85	1.16 ± 0.75	.930

a Values are expressed as n (%) or mean ± SD. unless otherwise indicated. Boldface indicates statistical significance. ICRS, International Cartilage Regeneration & Joint Preservation Society; JLCA, joint line convergence angle; mFTA, mechanical femorotibial angle; mHKAA, mechanical hip-knee-ankle angle; mLDFA, mechanical lateral distal femoral angle; mMPTA, mechanical medial proximal tibial angle.

There was no correlation between the amount of mechanical axis deviation (HKAA) and the volume of the OCD lesion or the ICRS grade on MRI scans. We found a correlation between the volume of the OCD lesion and the ICRS grade on MRI scans (*r* = 0.257; *P* < .001). The MRI ICRS grade was higher in patients reporting trauma before onset of symptoms (*P* < .001). Visibility on radiograph differed based on location: OCD on the patella or trochlea was visible in 66.7% of the cases, 4 of 6 and 6 of 9 cases, respectively. OCD on the LFC was visible in 85.5%, 53 of 62 cases. OCD on the MFC was visible in 91.3%, 146 of 160 cases. This did not reach statistical significance (*P* = .100).

**Figure 3.**
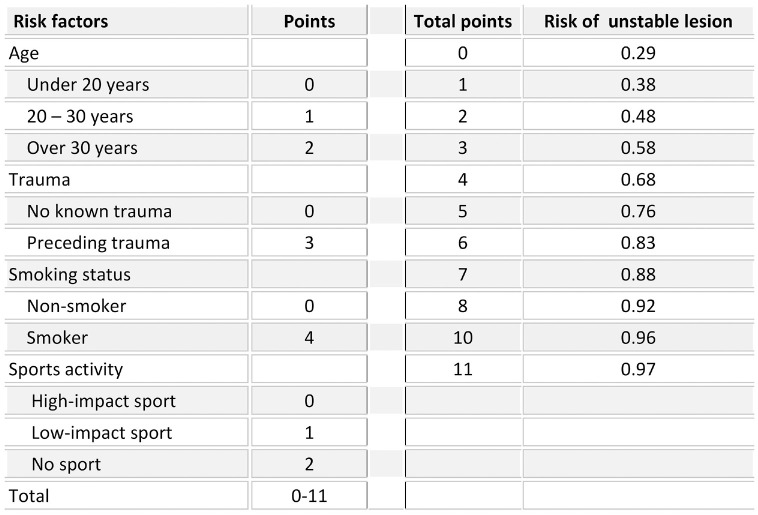
Risk factor calculator to predict osteochondritis dissecans lesion instability on magnetic resonance imaging.

**Figure 4.**
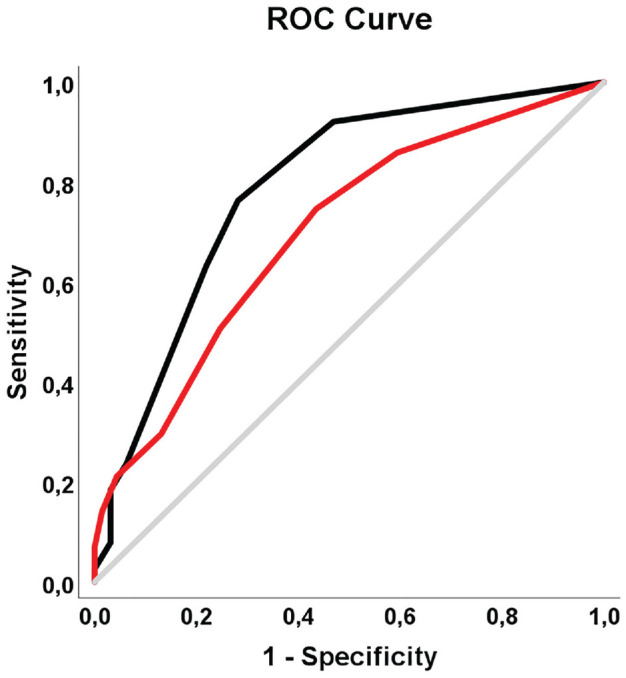
The area under the ROC curve was 0.70 (95% CI, 0.61-0.79) for the study group (middle line) and 0.79 (95% CI, 0.68-0.90) for the validation group (top line). ROC, receiver operating characteristic.

In 210 OCD lesions, stability was assessable on MRI. scans After randomization, the test group consisted of 140 lesions (69 stable, 71 unstable); the validation group, 70 lesions (32 stable, 38 unstable). In the prediction model developed in the study cohort, the risk of an unstable OCD lesion increased with age, active smoking status, no or low-impact sports participation, and preceding trauma. A multivariable scoring system (0-11 points) was generated, and an increased risk of lesion instability was seen with an increased score: 29.0% at 0 points and 97.0% at 11 points (Figure 3). The model, developed in the test group, produced an AUC of 0.70 (95% CI, 0.61-0.79). Internal validation within the validation group resulted in an increased AUC of 0.79 (95% CI, 0.68-0.90) (Figure 4).

## Discussion

This study provides detailed epidemiological data of 236 patients affected by OCD of the knee joint, a large OCD cohort. Our cohort presents patient characteristics that differ moderately from previously published cohort data, particularly in age, sex, and laterality for both skeletally mature and immature patients. We have shown that skeletally mature patients have a higher ICRS grade on MRI scans compared with skeletally immature patients, along with a higher mean BMI and a smaller number of patients practicing sports. For both skeletally mature and immature patients, we were further able to demonstrate a significant association between OCD of the MFC and varus malalignment and between OCD of the LFC and valgus malalignment. LFC lesions were also significantly associated with more valgus mechanical lateral distal femoral angle. Preceding trauma was significantly associated with a higher MRI ICRS grade at the time of initial presentation, whereas no association was found for mechanical axis deviation on MRI ICRS grade.

Our results differ considerably from current insights in patient characteristics and etiological factors. Patients in our cohort had a mean ± SD age of 21.8 ± 8.0 years when diagnosed with OCD of the knee joint. Although a mean of 3.3 ± 3.5 years had passed since first symptoms, this cohort is significantly older than most described in literature.^8,28,35^ Because only patients with written informed consent were included to this cohort, this might be due to adolescents being less likely to participate in research.^6^ It could also be due to previously published studies focusing mostly on pediatric patients and higher rates of medical coverage in young adults in the Dutch population. Despite our cohort consisting of more male than female patients (male to female ratio, 1.3:1.0) in both skeletally mature and immature patients, this was significantly lower than ratios found previously (range, 2.0:1.0 to 4.0:1.0).^20,22,28,35,39^ Because our cohort is fairly recent, a potential explanation is that OCD has become more common among adolescent girls as they become more active in sports in general and high-impact sports in particular.^46^ As the incidence of OCD has significantly increased in the 21st century,^26^ the patient characteristics are also likely to differ from those of earlier cohorts. For example, 9.8% of the patients in our cohort had bilateral lesions, whereas a range of 15% to 40% has been described in literature.^20^ Contralateral radiographs were not routinely obtained in clinical practice for asymptomatic patients, potentially missing asymptomatic OCD bilateral lesions. The MFC was affected in 66.4%, whereas the LFC was affected in 25.5%. Typically the MFC is affected in 70% to 85%, and the LFC is affected in 15% to 20% of the patients presented in literature.^3,18,20,22,35,39^ Our cohort had relatively more LFC lesions, although fewer OCD lesions were seen on the MFC. This could possibly be explained by the status of the study center as a tertiary referral hospital. LFC has been associated with a higher failure rate, specifically after operative treatment.^18,33,45,47^ As patients with previous unsatisfactory results are more likely to seek a second opinion, this might explain the larger numbers of patients with OCD in the LFC compartment. Another explanation might be the increased female population, generally associated with more valgus alignment.^32^

In our cohort, 6 of 236 (2.5%) patients had a medical history associated with osteonecrosis,^40^ equally distributed in skeletally mature and immature patients, which has not been reported before. Moreover, 5 of 236 (2.1%) patients had a congenital musculoskeletal disorder, including multiple epiphyseal dysplasia, spondyloepimetaphyseal dysplasia, congenital hip dysplasia, cerebral palsy, and spina bifida: 4 patients in the skeletally mature group and 1 patient in the skeletally immature group. As family history is not routinely examined in clinical practice, we have no data on hereditary or familial cause of OCD.

Furthermore, we found that skeletally mature patients had a higher ICRS grade on MRI scans than skeletally immature patients. Higher ICRS grades on MRI scans (grade 3 or 4) represent unstable lesions, which have low success rates when treated nonoperatively.^26^ This could add to the well-known paradigm that skeletal maturity is associated with a significantly decreased success rate for nonoperative treatment when compared with skeletal immaturity.^9,18,44^

We have also found that skeletally immature patients are more likely to participate in sports on a weekly basis, 86.1% compared with 72.6% in skeletally mature patients, and when practicing sports are more likely to practice high-impact sports, 83.8% and 57.9%, respectively. This age-based difference is also seen in the general Dutch population, although in both age groups, our cohort had a substantially higher percentage of sports participation. In the Dutch population, 74% of adolescents practice sports weekly compared with 56% of adults.^15^ Participation in high-impact sports has been previously associated with OCD of the knee.^2,18,26^ High levels of high-impact sports can lead to chronic mechanical overload of joints, and several studies have previously hypothesized this to be an important etiological factor for OCD.^5,16^

The mean time between onset of complaints and confirmed diagnosis was 3.3 ± 3.5 years, indicating a significant diagnostic prolongation. Such delayed presentation of patients with OCD was described by Cahill and Ahten,^9^ who reported that 80% of patients had complaints for 14 months before initial presentation. As many patients initially have few or vague complaints,^1,13,26^ routine imaging is not often performed. Given that in our cohort 12.0% of lesions were not visible on radiographs, this group may risk prolonged delay upon referral to a medical center. In this cohort, OCD of the patella and trochlea are less visible on plain radiographs, with only 66.7% being visible, compared with 85.5% on the LFC and 91.3% on the MFC. Although not statistically significant, this finding might warrant earlier use of MRI for assessment of OCD, especially in patients with patellofemoral complaints.

The current study highlights that the ICRS grade was significantly higher in patients who reported trauma before the onset of complaints. This is a new finding and has not been investigated in previous literature. This suggests that a preceding trauma is a risk factor for instability of an OCD lesion.

Our data further showed a statistically significant correlation between the leg axis and the location of the OCD lesion within the knee: 47.5% of patients (38/80) with OCD of the MFC had a varus leg alignment, associated with higher mechanical loading of the medial compartment, whereas 46.7% of patients (14/30) with OCD of the LFC had a valgus alignment, associated with higher mechanical loading of the lateral compartment (*P* = .002). Significant associations have been found previously,^7,19,23^ although generally higher correlations have been reported. Gonzalez-Harranz et al^19^ found that 70.0% of patients (28/40) with OCD of the MFC had a varus leg alignment, whereas 53.8% of patients (7/13) with OCD of the LFC had a valgus alignment. Brown et al^7^ found that 67.6% of patients (25/37) with OCD of the MFC had a varus leg alignment, whereas 66.7% of patients (16/24) with OCD of the LFC had a valgus alignment. We have also seen that patients with OCD lesions on the LFC had a statistically significantly lower mechanical lateral distal femoral angle (86.1°± 2.7°) compared with patients with OCD lesions on the MFC (88.5°± 2.7°), which suggests that patients with OCD lesions on the LFC have a more valgus-oriented distal femur compared with patients with OCD lesions on the MFC. This finding is less likely to be clinically significant, although it is consistent with the findings of Brown et al.^7^

To further explore the relation between OCD lesions and malalignment, we stratified the data to in situ and displaced OCD lesions. This revealed a similar correlation between the leg axis and the location of the OCD lesion for in situ lesions: 44.0% (22/50) of the OCD lesions of the MFC had a varus leg alignment, whereas 61.1% (11/18) of the OCD lesions of the LFC had a valgus leg alignment. Displaced lesions did not significantly differ; they showed varus leg alignment in 53.3% (8/15) of lesions on the MFC and valgus leg alignment in 33.3% (2/6) of lesions on the LFC. This supports the hypothesis that unfavorable leg malalignment is a contributing factor to OCD and not caused by displaced OCD lesions. However, displaced OCD lesions could exacerbate the degree of malalignment, specifically in lesions of the MFC. Mean mHKAA for in situ lesions was 1.42°± 2.52° varus compared with 3.08°± 4.78° varus in displaced OCD lesions (*P* = .214); mean mechanical femorotibial angle for in situ lesions was 1.26°± 2.52° varus compared with 3.60°± 4.09° varus in displaced OCD lesions (*P* = .050).

On the basis of our unique data, we have developed a model to predict lesion stability, applicable to the prehospital setting. Older age, active smoking status, inactivity in sports, and preceding trauma increase the risk of an unstable lesion. The finding that inactivity in sports increases the risk for unstable OCD lesions seems contradictory. We hypothesize that this is due to patients with higher sports activity experiencing restrictions sooner with vague complaints and poorly localized knee pain typically associated with stable lesions.^9^ In contrast, patients presenting with symptomatic lesions and not actively engaging in sports activity will be more likely to have unstable lesions, although no definitive statement can be made based on our data.

This model can potentially aid caregivers in assessing the urgency and necessity for referral to an orthopaedic surgeon specialized in operative techniques for OCD of the knee joint, especially when not readily available. With our internal validation, the model has been shown to be proficient. However, external validation is essential before clinical implementation should be considered.

Our study has several limitations. First, this is a retrospective study and is therefore subject to the inherent shortcomings of a retrospective study design. Full-leg standing leg-axis radiographs were available for only 113 of 259 affected knees. Second, because our department is a tertiary referral center, our cohort population consisted of both primary patients and secondary referrals. Because second opinions sometimes occur after unsatisfactory results of initial therapy, our patient characteristics could therefore be affected. Although baseline characteristics between primary and secondary referrals did not show significant differences, this should be considered when interpreting our data. Third, because our cohort population consisted of referred and diagnosed patients and was not taken from a general population, it therefore represents a biased population. Fourth, because patients had to give written consent, a total of 108 patients did not meet inclusion criteria, which is a risk for selection bias.

There are also several strengths of our study. First, this is one of the largest series of patients with OCD of the knee, where most of desired patient characteristics were available. This is, to the best of our knowledge, the largest cohort of patients assessed for axial alignment, with 110 affected femoral condyles with available full standing leg radiograph. Second, our cohort consisted of patients diagnosed with OCD of the knee, independent of potential treatment, and therefore patient characteristics were more representative, as opposed to cohorts describing only surgically treated patients.

## Conclusion

This study provides detailed epidemiological data of 236 patients affected by OCD of the knee. Older age, smoking, inactivity, and preceding trauma were predictive for instability of OCD lesions. We found an association between OCD of the MFC and varus malalignment and between OCD of the LFC and valgus malalignment. This, in combination with the high percentage of patients practicing high-impact sports, suggests an important role for mechanical overload in the pathogenesis of OCD.
